# Strength Gains as a Result of Brief, Infrequent Resistance Exercise in Older Adults

**DOI:** 10.1155/2014/731890

**Published:** 2014-09-30

**Authors:** James Fisher, James Steele, Pat McKinnon, Stephen McKinnon

**Affiliations:** ^1^Southampton Solent University, East Park Terrace, Southampton SO14 0YN, UK; ^2^Abstract Bodyworks, Precision Exercise, Newbury RG14 5BY, UK

## Abstract

Chronological aging is associated with a decrease in skeletal muscle mass and bone mineral density, an increase in fat mass, frequency of falls and fractures, and the likelihood of obesity, diabetes, and coronary heart disease. Resistance exercise has been shown to counter all of these effects of aging and, in turn, reduce the risk of all-cause mortality. However, variables such as volume and frequency have become contentious issues, with recent publications suggesting that similar physiological adaptations are possible with both high- and low-volume approaches. The aim of this research was to consider strength increases as a result of brief, infrequent resistance exercise. The present study offers data from 33 (14 male and 19 female) older adults (*M* = 55 years) who underwent brief (<15 minutes per exercise session), infrequent (2×/week), resistance exercise to a high intensity of effort (6-repetition maximum) at a controlled repetition duration (10 seconds concentric : 10 seconds eccentric) on 5 resistance machines (chest press, leg press, pull-down, seated row, and overhead press). Data is presented for training interventions of 12 weeks (male) and 19 weeks (female). Significant strength increases were identified for all exercises. With the detailed health benefits obtainable, the present study suggests that resistance exercise can be efficacious in much smaller volumes than previously considered.

## 1. Introduction

The natural homeostatic processes in the human body often result in a physical decline with age. We lose bone mineral density (BMD), muscle mass, and strength and we have an increase in fat mass, ultimately resulting in reduced physical performance [1–4]. As such, with aging there is generally an increased risk of acute and chronic conditions including greater frequency of bone fractures, obesity, diabetes, coronary heart disease, and cancers [5]. However, by performing resistance training (RT) a person can improve their strength [6], muscle size [7], cardiovascular fitness [8], metabolic health [9], and BMD [10]. As a result, people can decrease the potential for injuries through strengthening their joints, tendons, and ligaments [11, 12]. Hurley and Roth [13] comment that the data suggests that “*~2 decades of age-associated strength loss can be regained in ~2 months of resistance exercise.*” Indeed, reduced strength has been shown to be a strong risk factor for all-cause mortality independently of muscle mass [14]. Melov et al. [15] reported reversal in mitochondrial deterioration to the extent that participants with an average age of 68 years showed mitochondrial characteristics similar to those of persons with a mean age of 24 years following 6 months of resistance exercise. Succinctly, resistance exercise appears to reverse aging in skeletal muscle. Indeed, the evidence supports that resistance exercise reduces the risk of all-cause mortality [14–17].

Previous publications have suggested that greater loads result in greater increases in strength for older adults [18–21]. However, these studies failed to accurately control intensity of effort. Previous reviews in asymptomatic individuals of younger and middle age people have suggested that when intensity of effort is controlled, research does not support the superiority of a particular load and/or repetition range for increasing muscular strength [6] and size [7]. Other publications have discussed that differences in low and high loads can be equated in intensity of effort and thus negated by increasing repetition duration of low load training groups [22].

Research in young adults has supported this proposition showing similar strength and hypertrophic increases when using low loads (50–60% 1-repetition maximum (1 RM)) for longer repetition duration (3 seconds concentric : 1 second isometric : 3 seconds eccentric (3 : 1 : 3)) compared to higher loads (80–90% 1 RM) at shorter repetition durations (1 second concentric : 1 second eccentric (1 : 1)) [23, 24]. More recently van Roie et al. [25] have also reported nonsignificant differences in strength increases between low (20–40% 1 RM) and high (80% 1 RM) training loads in older adults when exercise is taken to a point of muscular failure. Certainly this is of important consideration since exercise ~1 RM produced an orthopaedic injury prevalence of ~20% in older adults [26]. In addition heavy loads/shorter repetition duration appear more likely to cause muscle soreness [27] which appears counterintuitive to persons wishing to improve their quality of life. We should also consider that near maximal loads are simply not representative of normal daily function.

Previous research has concluded that single sets of an exercise, performed to momentary muscular failure, produce similar strength gains to multiple sets [6, 28, 29]. This remains a contentious issue in the field [30]; however, there is limited research which has implemented and evaluated a single set approach with older adults. Indeed, a recent meta-analysis of resistance exercise in older adults [31] reported that all included studies used a multiple-set method. In fact, Westcott et al. [32] assessed a single set approach using 13 resistance exercises, 2-3×/week with older adults and reported significant strength increases favouring a group training at long repetition duration (10 seconds concentric : 4 seconds eccentric) compared to a group training at a more moderate repetition duration (2 seconds concentric : 1 second isometric : 4 seconds eccentric). Theoretically, moving a load more slowly (for a longer repetition duration) decreases the potential for external forces such as momentum to interact, thus maintaining muscular tension and likely increasing intensity of effort. Evidence supports that fewer repetitions are possible when moving a load at a longer-compared to shorter-repetition duration [33–35].

van Roie et al. [25] also considered the use of a single set protocol with older adults but limited training and testing to lower body exercises only. In an aging population with only 10–15% of persons over 55 years of age performing any strengthening activities [36] it is important to consider time-efficient methods which might encourage exercise adherence. The present authors have previously recommended single sets of an exercise, performed infrequently (1-2×/week), to a high intensity of effort, using resistance machines through a full range of motion, at a repetition duration that maintains muscular tension as being optimal for increasing strength whilst efficiently using time and minimising risk of injury [6, 37].

Whilst data from Westcott et al. [32] supports this approach, the present study represents a further decreased volume of training. The authors have worked closely with a UK exercise facility which uses these recommendations, categorically clarifying that all exercise sessions will be completed in <15 minutes, whilst stringently recording all workout data. As such, the present study aims to retrospectively present the data from the members of that facility emphasizing the ecological validity of* real people in a real gym*, rather than a “*laboratory gym*” in which most research is undertaken and restricted by specific protocols and research questions.

## 2. Methods

### 2.1. Study Design

This study was a retrospective analysis of strength outcomes of a cohort of members from a private UK based exercise facility. The facility uses standardised training protocols with members with all sessions being supervised by the same trainers who make meticulous records of every session allowing for analysis of load progression as a measurement of strength gains as a result of the training protocol administered. Participants training records were examined from the period beginning from January 2013 through to April 2014. The study design was approved by the relevant ethics committee at the author's institution.

### 2.2. Participants

Participants were required to have no medical condition for which RT is contraindicated to participate. Participant demographics are given in Table 1. Participants were existing members at the facility who provided written informed consent for their training data from their first session until their most recent to be released for analysis in this study. Power analysis of research using low volume RT in untrained participants was conducted to determine participant numbers (*n*) using an effect size (ES), calculated using Cohen's *d* [38] of ~1.0 [39] for the improvements in strength. Participant numbers were calculated using equations from Whitley and Ball [40] revealing a required 16 participants to meet required power of 0.8 at an alpha value of *P* ≤ 0.05 for detecting changes.

### 2.3. Equipment

Strength was measured using MedX (USA) torso arm (pull down), chest press, seated row, overhead press, and leg press resistance machines. These were also used for the RT intervention in addition to MedX (USA) leg extension, leg curl, bicep curl, torso flexion, hip extension, chest fly, seated dip, abdominal isolator, and lumbar extension resistance machines, as well as a pull-over (Nautilus, USA).

### 2.4. Participant Training

Throughout the time period analysed participants attended the facility to participate in supervised RT sessions ~2×/week. All participants performed a single set of torso arm (pull down), chest press, seated row, overhead press, and leg press exercises in this order throughout their training period and some occasionally performed 1-2 additional exercises using the other resistance machines noted. Each exercise was completed using a load that allowed the participants to perform a self-determined 6 RM (meaning that they determined inability to complete further repetitions if attempted, i.e., predicted momentary muscular failure on the next repetition) through a full range of motion using repetition duration of 10 seconds concentric and 10 seconds eccentric. This equated to total repetition duration of 20 seconds and a total time under load of ~120 seconds. The trainer monitored participants repetition duration throughout each exercise using a stopwatch and advised participants to either speed up or slow down as appropriate to maintain this repetition duration. Load progression was provided based on the following characteristics as assessed by the trainer; (1) the ability to maintain the prescribed repetition duration of 10 : 10 within a margin of 2 seconds error (i.e., 8–12 : 8–12), (2) the ability to maintain interrepetition consistency to this repetition duration within the set, (3) and the quality of the participants form for the exercise. Once the trainer was confident the participant could exceed a 6 RM whilst meeting these criteria with their current load, a further 2–5 lbs was added in their next training session. This method of progression is consistent with previous research [32]. The trainers throughout this intervention encouraged very strict form during exercise; for example, controlled and continuous breathing frequency (without a valsalva manoeuvre) and attempting to keep muscles which are not the target of the exercise as relaxed as possible.

As a time efficient training approach participants were also encouraged to move from one exercise to the next without significant rest, generally <30 seconds. All machines were prepared for the clients prior to beginning each exercise session to make this possible. With an average of 5 exercises per session, at ~120 seconds per exercise, total workout time is approximately 12 minutes. Indeed the trainers and the exercise facility specifically advertise that sessions will not exceed 15 minutes in total time commitment per training session. This represents an ecologically valid approach to applying the aforementioned recommendations with stringent, yet practical methods of increasing load.

Mean (± SD) numbers of training sessions are presented in Table 2 which equate to study duration of *M* = 12 ± 6.7 weeks for males and *M* = 19 ± 10.9 weeks for females. The SDs suggest large differences in actual duration between participants. However, this is likely representative of* real people*, where some people train for extended periods whilst others cease exercise intermittently as a result of other commitments.

### 2.5. Outcomes

Strength gains as progression in load used during exercise was the primary outcome for this study. As all participants had completed torso arm (pull down), chest press, seated row, overhead press, and leg press, load progression was examined for these exercises only. As participants continuously performed a standardised intervention, whereby the exercises were performed in the same order and used a self-determined 6 RM load (meaning that they determined inability to complete further repetitions if attempted that is, predicted momentary muscular failure on the next repetition) through a full range of motion using a repetition duration of 10 seconds concentric and 10 seconds eccentric throughout the training period, the increase in training load was considered to be adequate to determine strength gains as a result of the training completed. This was calculated as the training load in the most recent exercise session available for analysis minus the training load for the participants first training session.

### 2.6. Data Analysis

Training record data was available from 33 participants (male, *n* = 14; female, *n* = 19). Descriptive statistics including means and standard deviations were calculated for number of exercises performed each session, number of sessions completed for chest press, leg press, torso arm (pull down), seated row, and overhead press exercises, and load progression for these exercises. Data met assumptions of normality when examined using a Kolmogorov-Smirnov test. Gender comparisons were performed for demographic characteristics, number of exercises performed each session, number of sessions completed per exercise, and strength outcomes, including both absolute and relative change in training load and strength change relative to body mass, using an independent samples *t*-test. 95% confidence intervals (CI) were calculated in addition to ES using Cohen's *d* [38] for each absolute strength outcome to examine the significance and magnitude of effects where an outcome was considered to be significantly improved if the CI did not cross zero. Effect sizes (ESs) of 0.20–0.49 were considered as small, 0.50–0.79 as moderate and ≥0.80 as large.

## 3. Results

### 3.1. Participants

Participant baseline demographics are shown in Table 1. Age and BMI did not significantly differ between groups. Males had a significantly higher stature (*t*_(31)_ = 5.106, *P* < 0.001) and body mass (*t*_(29)_ = 2.983, *P* = 0.005) than females.

### 3.2. Training Sessions

Training session data including number of training sessions per exercise and exercises per sessions are presented in Table 2. Females had performed significantly more sessions than males for torso arm (*t*_(31)_ = −2.301, *P* = 0.028), seated row (*t*_(31)_ = −2.238, *P* = 0.033), and leg press (*t*_(30)_ = −2.126, *P* = 0.026) exercises. There was no difference in number of exercises performed per session between males and females.

### 3.3. Strength Outcomes

**Figure 1:**
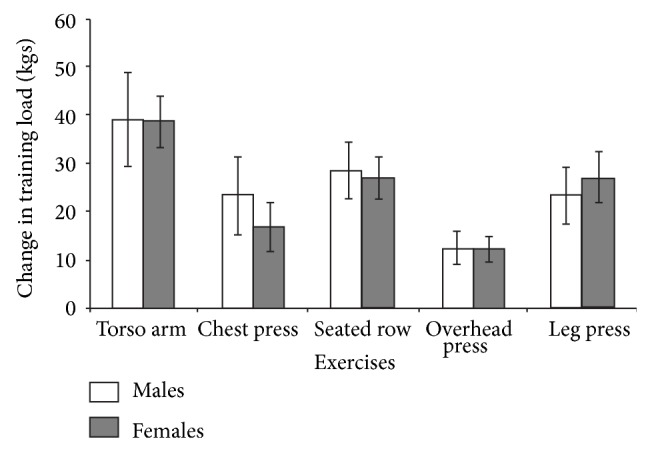
Mean change in absolute training load with 95% CIs for males and females.

**Figure 2:**
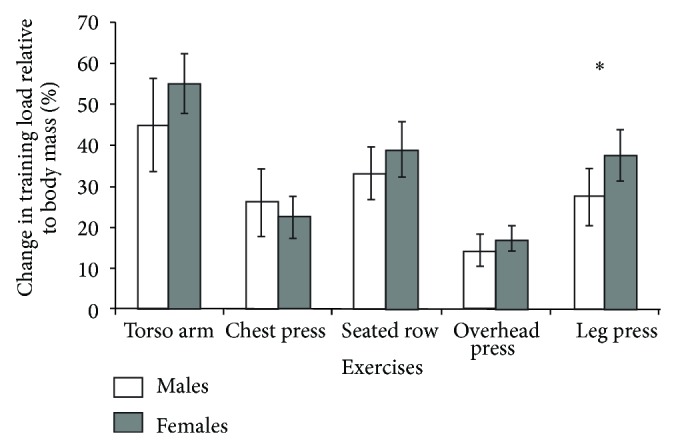
Mean change in training load relative to body mass with 95% CIs for males and females; ^*^significant compared to males (*P* < 0.05).

Beginning training loads are presented in Table 3. Males had a significantly higher absolute training load at baseline than females for torso arm (*t*_(31)_ = 3.488, *P* = 0.002), chest press (*t*_(31)_ = 4.215, *P* < 0.001), seated row (*t*_(30)_ = 2.603, *P* = 0.014), overhead press (*t*_(30)_ = 4.087, *P* < 0.001), and leg press (*t*_(30)_ = 3.898, *P* = 0.001) exercises. Strength relative to body mass did not differ at baseline between males and females for any exercise.  Figure 1 presents changes in absolute training load from first to last training sessions for each exercise for males and females. Change in absolute training load did not significantly differ between males and females for any exercise. 95% CIs suggest significant improvements in absolute strength for every exercise with large ESs for both males and females, respectively, of 2.14 and 3.31 for torso arm, 1.59 and 1.59 for chest press, 2.67 and 2.84 for seated row, 2.01 and 2.20 for overhead press, and 2.19 and 2.36 for leg press exercises. Relative increases in training load did not differ between males and females, respectively, for torso arm (68.7 ± 40.1% versus 90.8 ± 38.1%), chest press (55.8 ± 39.4% versus 59.0 ± 39.9%), seated row (65.0 ± 29.3 versus 81.2 ± 40.3%), and overhead press (39.0 ± 20.4% versus 58.0 ± 30.0%) exercises but was significantly greater for females for the leg press exercise (38.4 ± 18.2% versus 59.0 ± 28.6%; *t*_(30)_ = −2.297, *P* = 0.018).  Figure 2 presents changes in training load relative to body mass from first to last training sessions for each exercise for males and females. Changes in training load relative to body mass did not differ between genders for torso arm, chest press, seated row, or overhead press; however, they were significantly greater for females for the leg press exercise (*t*_(30)_ = −2.091, *P* = 0.045).

## 4. Discussion

This study presents data from a retrospective single arm trial of resistance training in older adults. Previous recommendations (e.g. [6]) have suggested single sets of an exercise to a high intensity of effort performed 1-2×/week as producing the same strength adaptations as larger training volumes/frequencies and yet presenting far greater time efficiency. Training interventions of similar methodology in a similar population sample have reported significant strength gains [32]. However, the present study examined an approach which used an average of two training sessions per week consisting of ~5 exercises to activate most muscle groups, equating to a total time commitment of approximately 30 minutes per week, a significantly lower volume of exercise than Westcott et al. [32]. Previous research has suggested that the addition of single-joint (SJ) to multijoint (MJ) exercises does not increase muscle hypertrophy beyond that of MJ exercises alone [41]. Further research has reported similar strength and hypertrophy increases when comparing SJ and MJ exercises independently [42]. The efficiency of performing only 5 exercises compared to larger volumes suggests practical benefits if the same adaptations are obtainable.

Participants within the present study showed significant meaningful increases in both absolute and relative to body mass strength (6 RM) as evidenced by 95% CIs (Figures 1 and 2) and large ESs for all exercises tested. Female participants reported similar increases in absolute load to male participants albeit with a greater number of training sessions, for example, a longer training duration. However, female participants also showed a significantly greater relative increase in strength, and increase relative to body mass, for the leg press exercise than males and qualitatively greater relative increases for all other exercises. Evidence has supported a greater magnitude of improvement in upper body compared to lower body strength between males and females [43] and also a potentially smaller age related decline in lower body strength and muscle quality in females compared to males [44]. However, there appears no prior evidence supporting the present data that females show greater relative increases in lower-body strength than males. We suggest these differences in relative strength increase may be a result of the significant strength differences at baseline between males and females and also that females engaged in a longer duration of training than males in the present study.

We have previously discussed that intensity of effort, and intent to maximally recruit muscle fibres appears to be the most significant variable affecting strength and hypertrophic increases (e.g., training to momentary muscular failure (MMF)) [6, 7]. However, the present data suggests that untrained older adults can make significant increases in strength by training to RM, which might best be thought of as volitional fatigue. Self-determined RM does not represent a quantifiable measure of intensity of effort as is evidenced by trained participants providing poor estimates at the number of repetitions possible before MMF [45]. As such, RM might not be scientifically meaningful regarding intensity of effort compared to MMF. However, training to volitional fatigue represents a very pragmatic approach, especially in the present population group. The data herein represents “*real people, doing real resistance exercise*” from which they are intending to acquire the aforementioned health and fitness benefits. We might surmise that their aims are to function more efficiently and for greater longevity in their day-to-day life. As the discomfort and debilitation associated with delayed onset muscle soreness (DOMS) which might arise as a result of high volume and/or very high intensity of effort (e.g., MMF) resistance exercise seems counterintuitive to a person wishing to have a more functional life.

Previous research suggests that perceived difficulty and misinformation about expected outcomes are barriers to older persons performing resistance exercise [36]. This study presents data from a UK based exercise facility where sessions are performed on a 1 : 1 basis (client : trainer). The study shows that resistance training need not be time consuming, dauntingly complex, or overly difficult, and that considerable increases in strength can be achieved. A potential limitation to this approach might be the financial expense and practicality of a 1 : 1 (trainer to client) session. Certainly the significant improvements seen within this intervention and other resistance training research might be a result of the individual coaching and motivation received by each participant. In considering transference from research to practical application, improvements to the same degree might not be possible in most health clubs/gyms and so forth, where this ratio is expensive/inappropriate. However, future research might consider the efficacy of small group resistance exercise sessions (e.g., 2–5 participants : 1 trainer). Previous research has shown significant improvements in function as a result of group exercise (*n* = ~23 and *n* = 16–20 persons) in studies where mean age = 65 years [46] and 74 years [47]. However, Gentil and Bottaro [48] reported greater increases in upper and lower body strength in high supervision (1 : 5; trainer to athlete ratio) compared to a low supervision (1 : 25) group. Certainly improvements to the magnitude shown within the present study are possible from such a low frequency and volume of training suggests that there is scope to further evaluate this approach.

In the interests of transparency we have previously discussed that publishing data which does not identify control/clarity of variables potentially offers little to trainers or trainees with regard to how they might optimise training adaptations [49]. However, the protocol reported herein is highly standardised between participants and we offer the present findings to highlight the concept of undertaking this protocol and similar ones given the ecological validity of the study.

In summary our data suggests that when training to RM significant strength increases are possible from brief (<15 minutes/~5 exercises per workout), infrequent 1-2×/week, resistance exercise sessions. As previous research has indicated that strength is an independent risk factor for all-cause mortality [14], these results are meaningful for reducing this risk in the population examined. Previous research has shown that resistance exercise in older adults can significantly increase strength, muscle mass, and bone mineral density, improve gene expression and mitochondrial characteristics, and reduce the risk of falls, obesity, and type 2 diabetes and, as noted, reduce the risk of all-cause mortality. Since the present data suggest that strength can be significantly increased by following the aforementioned protocol, future research should consider whether other health markers such as blood pressure and glycemic control respond to the same low volume stimulus.

## Figures and Tables

**Table 1:** Participant demographic characteristics (Mean ± SD).

	Males	Females
Age (years)	55 ± 10	55 ± 11
Stature (cm)	177.6 ± 5.5	167.4 ± 5.8
Body mass (kg)	85.92 ± 12.50	71.43 ± 13.56
BMI	27.54 ± 4.11	25.63 ± 5.50

**Table 2:** Participant training session data.

Exercise	Number of training sessions (*M* ± SD)	
	Males	Females
Torso arm (pull down)	23 ± 12	37 ± 21∗
Chest press	25 ± 16	40 ± 24
Seated row	24 ± 12	39 ± 23∗
Overhead press	24 ± 12	34 ± 16
Leg press	24 ± 15	41 ± 25∗
Exercises per session (number)	5 ± 1	5 ± 1

^*^Significant compared to males (*P* < 0.05).

**Table 3:** Beginning training loads.

	Males	Females
Mean (±SD) training load (Kgs)		
Torso arm	61.23 ± 14.54	45.36 ± 10.34∗
Chest press	43.16 ± 10.80	29.98 ± 7.17∗
Seated row	47.66 ± 14.87	36.48 ± 9.48∗
Overhead press	33.29 ± 8.02	22.92 ± 6.31∗
Leg press	63.50 ± 13.35	47.56 ± 7.58∗

^*^Significant compared to males (*P* < 0.05).
